# Host genotype, resistance status and seasonal temperature shape the Norway spruce phyllosphere mycobiome

**DOI:** 10.1371/journal.pone.0360930

**Published:** 2026-10-23

**Authors:** Wenzi Ren, Zilan Wen, Kai Wang, Abiodun Abeeb Azeez, Fred O. Asiegbu

**Affiliations:** 1 Department of Forest Sciences, University of Helsinki, Helsinki, Finland; 2 College of Forestry, Fujian Agriculture and Forestry University, Fuzhou, China; University of Salento: Universita del Salento, ITALY

## Abstract

The influence of the phyllosphere microbiome on plant health and disease development is well established. However, the roles of host resistance phenotype, seasonal variations, and accumulated temperature in shaping the phyllosphere mycobiome of Norway spruce (*Picea abies*) remain poorly understood. In this study, we investigated (i) the phyllosphere mycobiome assembly of a subset of *Heterobasidion* partially resistant and susceptible Norway spruce genotypes, (ii) how fungal community composition in these genotypes changes across seasons and with temperature, and (iii) the extent to which the phyllosphere mycobiome shared taxa with the seed-associated fungal communities of the same genotypes. Needle samples were collected from *Heterobasidion* partially resistant and susceptible Norway spruce seedlings growing in an open field in May, July and September 2022. Fungal communities were characterized using internal transcribed spacer (ITS) amplicon sequencing. Alpha diversity metrics (richness, diversity and evenness) of the phyllosphere fungal community were significantly different between susceptible and partially resistant Norway spruce genotypes. Although the distributions showed substantial overlap between resistance classes, all three alpha-diversity metrics differed significantly between the groups (p < 0.05). Community composition was associated with both host resistance phenotype and temperature-related variables, with average and accumulated temperature explaining a greater proportion of variation than host resistance phenotype. Less than 1% of phyllosphere fungal taxa overlapped with the seed-associated fungal communities. Network analyses further revealed seasonal differences in fungal association patterns between partially resistant and susceptible seedlings.

## 1. Introduction

Plant-associated microbiomes ubiquitously colonize above- and below-ground tissues as epiphytes or endophytes and play fundamental roles in plant growth, nutrient acquisition, stress tolerance, immunity, and disease resistance [1–3]. These intimate associations have led to the concept of the plant microbiome as an extension of the host genome [4], highlighting its importance in plant adaptation to changing environments. Consequently, understanding the factors that shape plant-associated microbial communities has become a major focus of plant and microbial ecology [5,6].

Plant microbiome assembly is governed by complex interactions between host genetic factors and environmental conditions. Host genotype shapes microbial recruitment by modulating immune responses, tissue chemistry, phytohormone signaling, and the production of specialized metabolites, which together create selective filters for colonizing microbes [7–10]. Environmental factors, including temperature, moisture, pH, and oxygen availability, further shape microbial colonization both directly and indirectly through their effects on plant physiology [11]. These interacting processes are expected to be particularly important in above-ground plant tissues, where microbial communities experience highly dynamic environmental conditions.

Research on plant microbiomes has largely focused on below-ground communities associated with the rhizosphere and rhizoplane because of their fundamental roles in nutrient acquisition, growth, defence and overall health and productivity [12–15]. More recently, increasing attention has been directed towards the phyllosphere microbiome, which inhabits one of the largest microbial habitats on Earth [7,16–18]. Unlike below-ground environments, the phyllosphere experiences pronounced fluctuations in temperature, humidity and solar radiation, making seasonal environmental conditions major drivers of microbial community assembly [19]. Despite these dynamic conditions, phyllosphere microbial communities contribute to plant health by enhancing pathogen suppression and tolerance to abiotic stress [18,20,21]. In addition to environmental filtering, host genetic variation appears to exert a stronger influence on phyllosphere microbial communities than on rhizosphere communities [8,9], highlighting the phyllosphere as an excellent system for investigating how host genetic background shapes microbial community assembly.

Norway spruce (*Picea abies*) is an ecologically and economically important conifer tree species, broadly distributed across the northern hemisphere [22]. However, its productivity is threatened by *Heterobasidion* species, one of the most economically damaging diseases affecting European conifer forests accounting for annual losses estimated at approximately €800 million across Europe, including €50–80 million in Finland alone [23]. Climate change is expected to increase disease incidence and severity by creating environmental conditions more favourable for pathogen development and host infection [24–26]. Although considerable efforts have been devoted to disease management, current control strategies remain only partially effective. Previous studies have demonstrated that Norway spruce genotypes differing in resistance to *Heterobasidion* exhibit distinct molecular and physiological responses in needle tissues [27–29], indicating that above-ground tissues reflect systemic host responses associated with disease resistance. However, whether genetically determined differences in disease resistance are associated with variation in phyllosphere fungal communities remains largely unknown. Therefore, understanding how host genotype influences phyllosphere microbiome assembly could provide new opportunities for improving disease management and forest tree breeding [9]. Beneficial microbial taxa associated with resistant genotypes may contribute to pathogen suppression, whereas identifying host genetic traits that shape microbial community assembly may facilitate the development of microbiome-informed selection strategies [10,30]. Furthermore, using a culture-free molecular approach, Rajala et al [31] showed that host genotype influences the abundance and community composition, but not richness, of Norway spruce needle endophytes. The authors also examined needle endophytes in Norway spruce cuttings from slow- and fast-growing clonal origins. Although endophyte communities did not differ between clonal origins, they correlated with current cutting growth, suggesting links to short-term host performance [32]. Overall, they concluded that endophyte communities were shaped by both host identity and environmental factors.

Despite growing interest in plant microbiomes and increasing recognition of the seasonal dynamics of phyllosphere microbial communities [17], relatively few studies have investigated how host genetic background interacts with seasonal environmental variation to shape phyllosphere fungal communities in forest trees. In particular, the influence of accumulated seasonal temperature, an ecologically relevant measure of thermal exposure that integrates microbial growth conditions over time, remains poorly understood. Furthermore, the contribution of seed-associated fungi to phyllosphere microbiome assembly in long-lived forest trees has received little attention. Here, we investigated how host genotype and accumulated seasonal temperature jointly influence the phyllosphere mycobiome of Norway spruce genotypes exhibiting contrasting resistance to *Heterobasidion parviporum.* We further examined the overlap between seed-associated and needle-associated fungal communities to evaluate the potential contribution of vertically transmitted fungi to phyllosphere microbiome assembly. We hypothesized that (i) Norway spruce genotypes differing in resistance to *H. parviporum* harbour distinct phyllosphere fungal communities, (ii) accumulated seasonal temperature is a major driver of temporal variation in community assembly, and (iii) seed-associated fungi contribute only a limited proportion of the fungal taxa detected in seedling needles.

## 2. Materials and Methods

### 2.1 *Heterobasidion*-partially resistant and susceptible Norway spruce seedlings

Norway spruce genotypes exhibiting contrasting resistance to *Heterobasidion parviporum* were selected based on lesion-length phenotypes and survival rates previously characterized in a greenhouse inoculation experiment [33]. Briefly, 402 two-year-old open-pollinated Norway spruce seedlings were inoculated with *H. parviporum* isolate 04002 under greenhouse conditions between February and June 2021. Four months after inoculation, lesion lengths on the xylem surface were measured to assess disease severity. Across the screened population, lesion lengths ranged from 6.3 ± 0.75 mm to 15.1 ± 2.2 mm (mean ± SD). Seedlings (half-sibs) with survival rates below 33% were excluded from further consideration to avoid selecting genotypes with poor post-inoculation survival. Best linear unbiased predictions (BLUPs) of lesion length were then estimated using linear mixed-effects models that accounted for clonal, family, block and pathogen-batch effects. The remaining seedlings were ranked according to their BLUP-estimated lesion lengths, with lower values indicating greater resistance. Five half-sib genotypes with the lowest BLUP-estimated lesion lengths were classified as partially resistant (PR), whereas another five half-sib genotypes with the highest BLUP-estimated lesion lengths were classified as susceptible (S), resulting in a total of 10 selected genotypes [33]. Four biological replicates of each selected genotype were subsequently planted at the Viikki Experimental Field in Helsinki, Finland, in August 2021. The field site had previously been cultivated with birch seedlings in 2019 and with oat and barley in 2020. To minimize potential neighbourhood effects, seedlings were planted at 1-m intervals with alternating PR and S half-sib seedlings (S1 Fig).

Needle samples were collected from all four field replicates of each selected genotype on 5 May, 7 July and 30 September 2022 to characterize temporal variation in the phyllosphere mycobiome. For each biological replicate, three needles were collected and pooled to constitute one sample, resulting in 120 needle samples across the three sampling periods. The average daily temperatures on the sampling dates were 6℃, 16.6℃ and 10℃, respectively (AccuWeather; https://www.accuweather.com/). The accumulated temperature during the 60 days preceding each sampling date was 120.0℃, 785.5℃ and 901.5℃, respectively (S2 Fig). Following collection, needle samples were immediately frozen in liquid nitrogen and subsequently stored at −80℃ until DNA extraction. For comparison with seed-associated fungal communities, 0.5 g of seeds was sampled from the same seed lots supplied by the Natural Resources Institute Finland (Luke) that were used to raise the half-sib genotypes.

### 2.2 DNA extraction, amplification of the ITS region, and sequencing

Needles were not washed or surface sterilized prior to DNA extraction to retain both epiphytic and endophytic fungal communities, which together were considered the phyllosphere mycobiome. Needle samples were homogenized in liquid nitrogen [34], and genomic DNA was extracted using a modified cetyltrimethylammonium bromide (CTAB) protocol [35,36]. The homogenized needle tissue was transferred to a 2-mL microcentrifuge tube containing 900 μL preheated CTAB extraction buffer and incubated according to the modified protocol. Following CTAB extraction, the aqueous phase was purified by two successive extractions with chloroform: isoamyl alcohol (24:1, v/v). For each extraction, 900 μL chloroform: isoamyl alcohol was added, mixed thoroughly, and centrifuged, after which the aqueous phase was transferred to a fresh tube. DNA was subsequently precipitated, washed with 70% ethanol, and resuspended in nuclease-free water. DNA concentration and purity were assessed using a NanoDrop ND-1000 spectrophotometer (Thermo Fisher Scientific, USA). PCR amplification and sequencing of the fungal ITS2 region were performed by Novogene (Cambridge Science Park, UK). Amplicons were generated using the primer pair ITS3-2024F (5′-GCATCGATGAAGAACGCAGC-3′) and ITS4-2409R (5′-TCCTCCGCTTATTGATATGC-3′). PCR products were purified and sequenced on an Illumina paired-end platform, generating 250-bp paired-end reads. Raw sequence data have been deposited in the National Center for Biotechnology Information (NCBI) Sequence Read Archive (SRA) under BioProject accession number PRJNA1072281.

### 2.3 Bioinformatics

Raw paired-end ITS2 sequences were demultiplexed, and primer sequences were removed by Novogene UK using Cutadapt. Sequence quality control, denoising, paired end read merging, and chimera removal were performed in QIIME2 version 2023.9 with the DADA2 plugin [37]. Based on the quality profiles of the reads, forward and reverse sequences were truncated at 220 bp and 178 bp, respectively, to retain high-quality bases (Phred quality score >25). Taxonomic assignment was performed against the UNITE fungal release for Fungi 2 (2022) reference database [38]. Amplicon sequence variants (ASVs) represented by fewer than 10 reads and ASVs not assigned to the kingdom were removed prior to downstream analyses. Based on the rarefaction curves, all samples were rarefied to 30,805 reads per sample, which provided a common sequencing depth across samples and sufficient sampling coverage for downstream community analyses. Rarefaction was selected to minimize the potential influence of unequal sequencing depth on comparisons among samples. The resulting rarefied dataset comprised 3,854 ASVs and was used for subsequent downstream analyses. Alpha-diversity metrics (Shannon diversity, observed richness and Pielou’s evenness) were calculated in QIIME2 to assess fungal community diversity, richness, and evenness. Subsequent statistical analyses and data visualization were performed in RStudio.

Alpha-diversity metrics were visualized using the ggpubr package [v0.6.0] [39]. The relative abundances of fungal taxa and network properties were visualized using ggplot2 [v3.4.2] [40]. Data normality and homogeneity of variance were assessed using the Shapiro–Wilk and Levene’s tests, respectively, prior to parametric comparisons. One-way analysis of variance (ANOVA) was used to evaluate differences among sampling periods, while Student’s *t*-tests were used to compare partially resistant (PR) and susceptible (S) genotypes when the assumptions of normality and homogeneity of variance were satisfied. Beta diversity was assessed using Bray–Curtis dissimilarities based on calculated ASV relative abundances. Principal coordinates analysis (PCoA) was performed using the vegan package [v2.6-4] [41], and visualized with ggplot2. Permutational multivariate analysis of variance (PERMANOVA) was conducted using the vegan package to evaluate differences in fungal community composition among sampling periods and between resistance classes. Fungal ecological guilds were assigned using FUNGuild [42]. Guild composition was visualized using ggplot2, and differences in guild relative abundances between PR and S survival were assessed using Student’s t-tests when the assumptions of normality and homogeneity of variance were satisfied.

Microbial association networks were constructed based on Spearman rank correlations among ASVs. To reduce sparsity, only ASVs accounting for 90% of the total sequence abundance were retained. Correlations with Spearman’s ρ > 0.6 and *P* < 0.05 were considered significant and used for network construction. Network degree distributions were visualized using ggpubr.

Distance-based redundancy analysis (db-RDA) was performed using the vegan package to assess relationships between fungal community composition and environmental variables. ASVs with relative abundances greater than 0.1% were included in correlation analyses with environmental factors. Correlation patterns were visualized using the pheatmap package [v1.0.12] [43]. The potential contribution of seed-associated fungal communities to the phyllosphere mycobiome was evaluated using SourceTracker [v0.9.1] [44], with seed samples treated as source communities and needle samples treated as sink communities.

## 3. Results

### 3.1 Fungal assemblages in partially resistant and susceptible Norway spruce phyllospheres

The richness, diversity and evenness of the phyllosphere fungal community differed significantly between susceptible and partially resistant Norway spruce genotypes (Fig 1a). In contrast, principal coordinate analysis (PCoA) revealed a separation between phyllosphere and seed fungal communities, whereas the phyllosphere fungal communities of susceptible and partially resistant genotypes largely overlapped (Fig 1b). The first two PCoA axes explained 47.1% of the total variation, indicating that the two-dimensional ordination captured less than half of the variation in fungal community composition. This pattern was further supported by PERMANOVA, which detected significant differences between seed and phyllosphere communities but not between the phyllosphere communities of susceptible and partially resistant genotypes.

**Fig 1:**
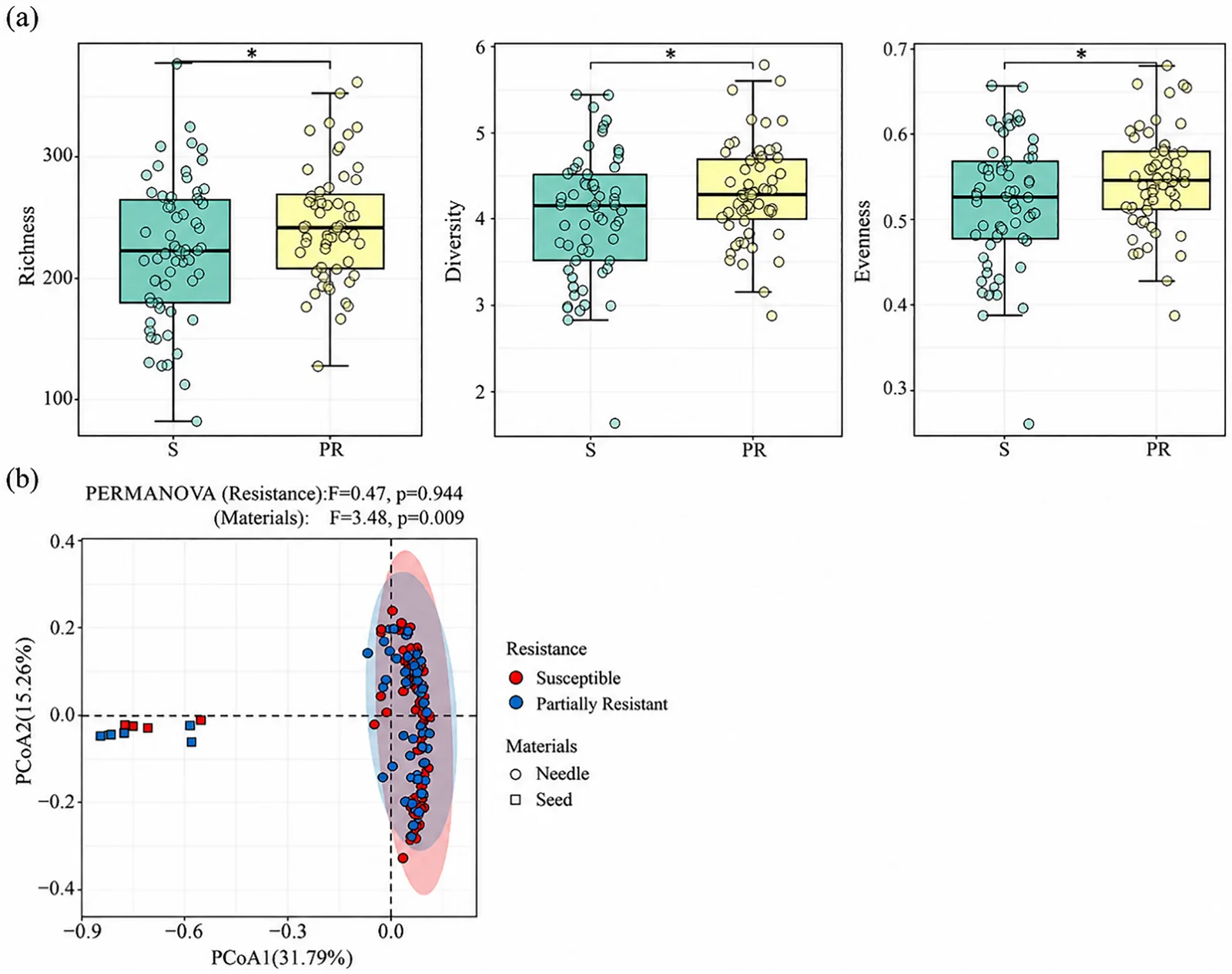
(a) Fungal alpha diversity in *Heterobasidion* tolerant and susceptible Norway spruce needles. S: susceptible genotypes, PR: partially resistant genotypes. (b) Principal Coordinates Analysis of Bray-Curtis distance of phyllosphere and seed samples.

### 3.2 Seasonal variation in the phyllosphere fungal communities of partially resistant and susceptible seedlings

Seasonal changes in phyllosphere fungal community diversity differed between partially resistant and susceptible Norway spruce genotypes. In susceptible genotypes, alpha diversity indices were significantly lower in September than in May or July, whereas no significant seasonal changes were observed in partially resistant genotypes (Fig 2a). Consequently, alpha diversity differed significantly between partially resistant and susceptible genotypes in September but not in May or July (S3 Fig). The PCoA indicated temporal variation in fungal community composition from May to September in both partially resistant and susceptible genotypes. PERMANOVA further supported a significant effect of sampling period on fungal community composition (Fig 2b).

**Fig 2:**
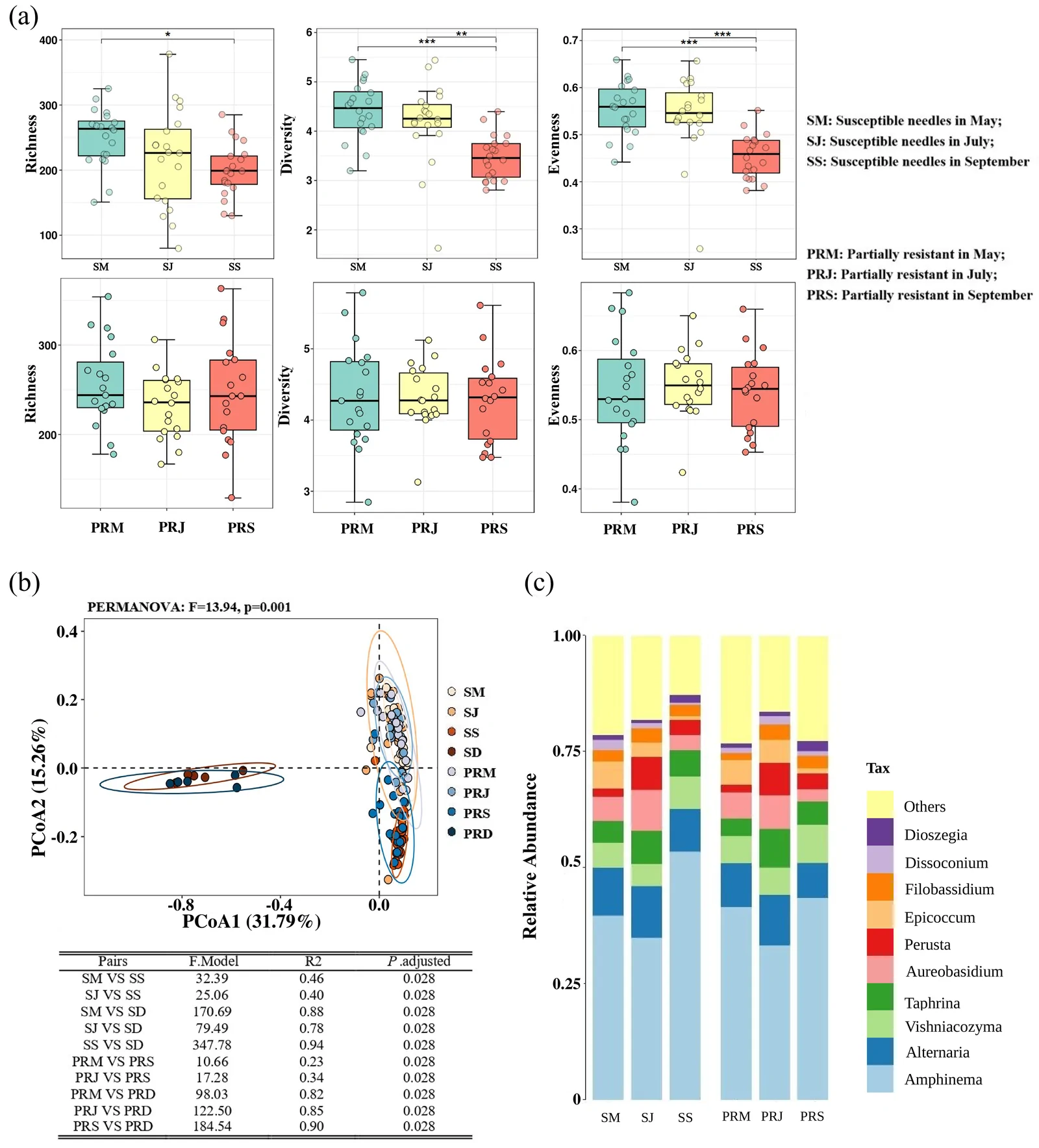
Seasonal impacts on *Heterobasidion*-partially resistant and susceptible Norway spruce phyllosphere microbiome assembly. (a) Seasonal impact on microbiome alpha diversity; (b) Principal Coordinates Analysis of samples from different seasons. Pairwise PERMANOVA analyses were performed between different seasons and between seed– phyllosphere comparison. SD: susceptible seed, PRD: Partially resistant seed; (c) Relative abundance of phyllosphere microbiome from different seasons at the genus level.

The ten most abundant fungal genera in the Norway spruce phyllosphere were *Amphinema*, *Alternaria*, *Vishniacozyma*, *Taphrina*, *Aureobasidium*, *Perusta*, *Epicoccum*, *Filobasidium*, *Dissoconium* and *Dioszegia* (Fig 2c). The relative abundances of *Epicoccum*, *Amphinema*, *Vishniacozyma* and *Aureobasidium* changed significantly across the growing season in both susceptible and partially resistant genotypes (S1 Table). Compared to partially resistant phenotypes, *Filobasidium* was significantly more abundant in susceptible genotypes in May (*P* = 0.03), whereas *Amphinema* was significantly more abundant in susceptible genotypes in September (*P* < 0.001). However, no significant differences in abundance of taxa were detected in partially resistant phenotypes in July (S1 Table).

### 3.3 Seasonal dynamics of phyllosphere fungal functional guilds

The four most abundant fungal functional guilds in the phyllosphere of both susceptible and partially resistant Norway spruce genotypes were mutualistic symbionts, plant pathogens, undefined saprotrophs, and endophytes (Fig 3). The remaining dominant guilds comprised animal pathogens, wood saprotrophs, fungal parasites, epiphytes, lichen parasites, and plant saprotrophs. Significant differences in the relative abundances of functional guilds between susceptible and partially resistant genotypes were detected only in September, primarily for mutualistic symbionts and undefined saprotrophs (*P* < 0.05). *Amphinema* was the predominant genus assigned to the mutualistic symbiont guild (S2 Table).

**Fig 3:**
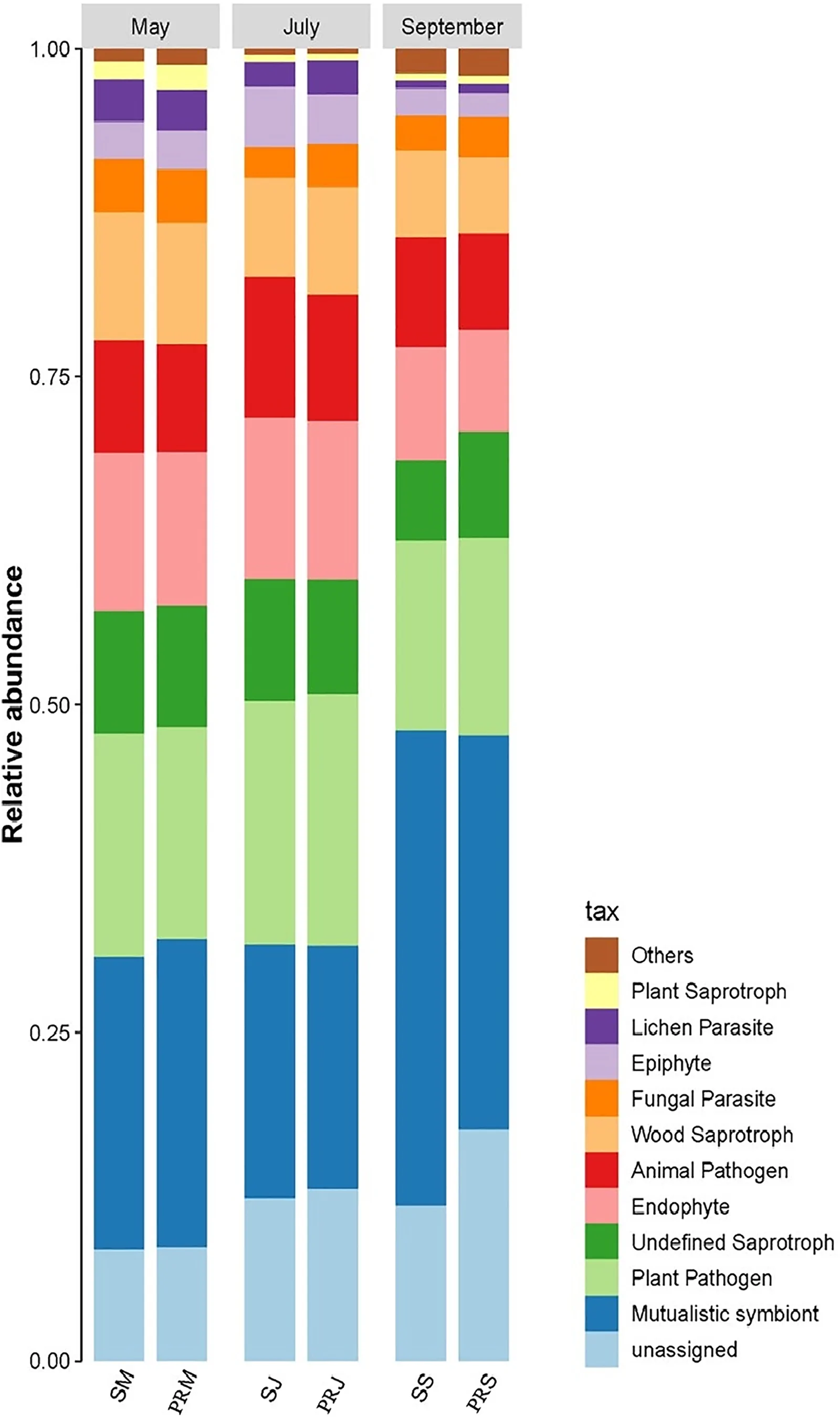
Predicted functional composition of susceptible and partially resistant Norway spruce phyllosphere microbiome in May, July and September.

### 3.4 Ecological association networks of phyllosphere fungal communities

The complexity of the phyllosphere fungal co-occurrence networks increased over the growing season in both partially resistant and susceptible Norway spruce genotypes, as indicated by an increasing number of network edges from May to September (Fig 4a). Susceptible genotypes exhibited more network edges than partially resistant genotypes in May and July, whereas the opposite pattern was observed in September. The number of negative associations was lowest in July and highest in September for both genotype groups (Fig 4a).

**Fig 4:**
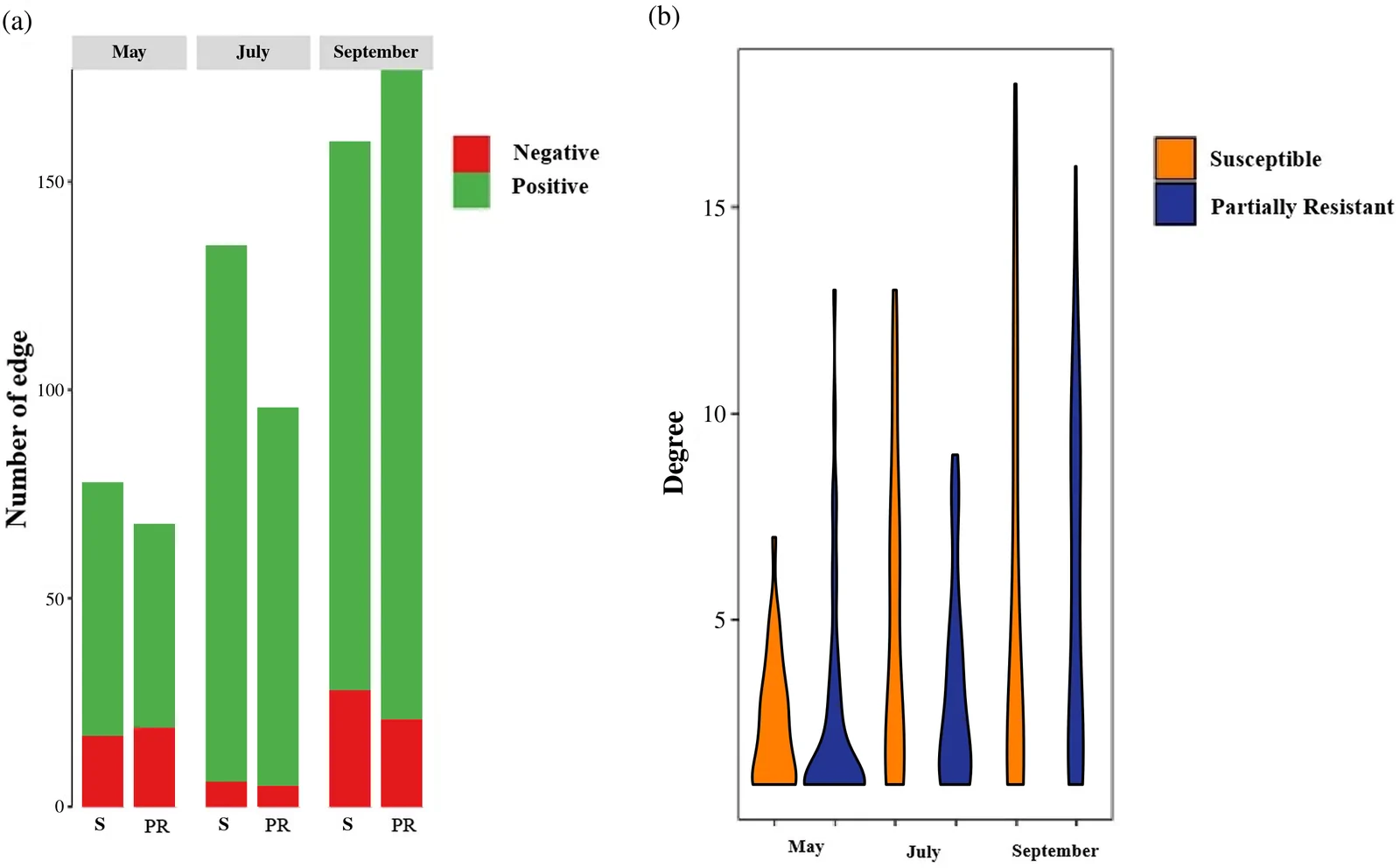
Ecological correlation network topology values. (a) Edge bar plot of susceptible and Partially resistant genotypes. Negative edge indicates that the connected microbes had different ecological niches or were antagonistic to each other. Positive edge indicates that the connected microbes had a similar niche or were mutualistic to each other; (b) Degree distribution of susceptible and partially resistant genotypes.

Node degree, which reflects the number of connections associated with each ASV, generally increased from May to September in susceptible genotypes. In contrast, node degree fluctuated seasonally in partially resistant genotypes without a consistent temporal trend (Fig 4b).

### 3.5 Composition, functional guilds and overlap between seed and phyllosphere fungal communities of Norway spruce

Five of the ten most abundant fungal genera were shared between seed and phyllosphere communities. However, their relative abundances differed between the two tissues. The three most abundant genera in seeds were *Sirococcus*, *Hormonema*, and *Kendrickiella* (Fig 5a). Seed fungal community composition did not differ significantly between partially resistant and susceptible genotypes (S3 Table).

**Fig 5:**
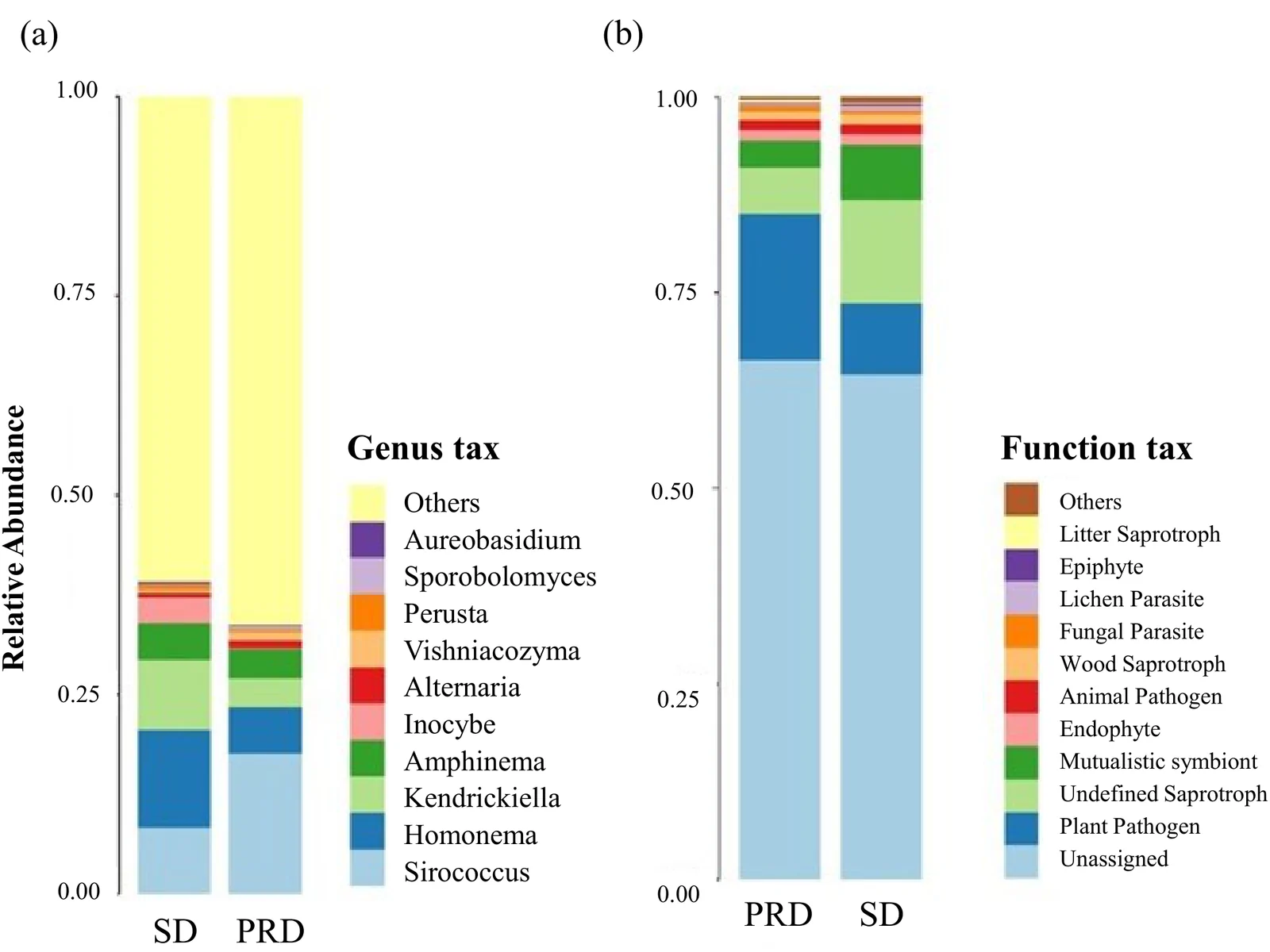
*Heterobasidion* partially resistant and susceptible Norway spruce seed mycobiome composition of taxonomy and function. (a) Seed species composition at the genus level; (b) Seed function composition. SD: susceptible seed, PRD: Partially resistant seed.

More than 60% of fungal taxa detected in seeds could not be assigned to a functional guild (Fig 5b). Among the classified taxa, plant pathogens, undefined saprotrophs and mutualistic symbionts were the dominant guilds in both partially resistant and susceptible seeds. Plant pathogens were the most abundant guild in partially resistant seeds, whereas undefined saprotrophs predominated in susceptible seeds. However, the relative abundances of functional guilds did not differ significantly between resistance phenotypes (*P* > 0.05; S4 Table).

SourceTracker analysis estimated that seed-associated fungi contributed only 0.57% and 0.18% of the phyllosphere fungal communities in partially resistant and susceptible genotypes, respectively (Table 1). More than 94% of the phyllosphere fungal community was shared among seedling phyllosphere samples, whereas more than 5% originated from unidentified sources. These proportions did not differ significantly between resistance phenotypes (*P* > 0.05).

**Table 1:** Analysis of origins of Norway spruce phyllosphere microbiome source.

Tolerant level	Sample ID	Needle	Seed	Unknown
Susceptible	SJ157.1	92.79%	0.04%	7.17%
	SJ164.1	96.51%	0.16%	3.33%
	SJ291.1	92.48%	0.22%	7.30%
	SJ395.1	96.70%	0.37%	2.93%
	SJ92.1	79.89%	0.16%	19.95%
	SM157.1	96.96%	0.08%	2.96%
	SM164.1	97.90%	0.12%	1.98%
	SM291.1	94.52%	0.21%	5.27%
	SM395.1	98.37%	0.04%	1.59%
	SM92.1	88.62%	0.12%	11.26%
	SS157.1	98.32%	0.07%	1.61%
	SS164.1	90.28%	0.08%	9.64%
	SS291.1	99.75%	0.08%	0.17%
	SS395.1	99.03%	0.67%	0.30%
	SS92.1	98.53%	0.32%	1.15%
	Average	94.71%	0.18%	5.11%
Partially resistant	PRJ3.1	98.72%	0.09%	1.19%
	PRJ345.1	96.99%	0.16%	2.85%
	PRJ402.1	95.64%	0.15%	4.21%
	PRJ56.1	94.48%	3.10%	2.42%
	PRJ67.1	96.69%	0.22%	3.09%
	PRM3.1	98.94%	0.17%	0.89%
	PRM345.1	97.58%	0.15%	2.27%
	PRM402.1	96.97%	0.14%	2.89%
	PRM56.1	75.68%	0.08%	24.24%
	PRM67.1	80.79%	0.14%	19.07%
	PRS3.1	98.76%	0.11%	1.13%
	PRS345.1	98.00%	0.07%	1.93%
	PRS402.1	91.33%	2.92%	5.75%
	PRS56.1	98.37%	0.61%	1.02%
	PRS67.1	91.81%	0.49%	7.70%
	Average	94.05%	0.57%	5.38%

Despite the limited overall contribution of the seed mycobiome, a greater number of seed-associated taxa were detected in the phyllosphere of partially resistant genotypes than in susceptible genotypes, particularly in September (Tables 1 and 2). Six shared taxa from partially resistant genotypes were identified to the species level: *Pyrenopeziza revincta* (May), *Dissoconium nelumbonis* (July), and *Dothiora coronillae*, *Dissoconium proteae*, *Cystobasidium pinicola*, and *Pachyramichloridium pini* (September).

**Table 2:** Unique Norway spruce phyllosphere microbiome transferred from seeds.

	Susceptible		Partially resistant	
	Sum	Unique taxonomy	Sum	Unique taxonomy
M	20	–	19	*Pyrenopeziza revincta*
				f Mycosphaerellaceae
J	23	g *Aureobasidium*	23	o Xylariales
		*Ramularia stellenboschensis*		*Dissoconium nelumbonis*
		o Tremellales		
S	21	g *Phaeosphaeria*	31	*Dothiora coronillae*
				*Dissoconium proteae*
				*Cystobasidium pinicola*
				o Myriangiales
				*Pachyramichloridium pini*
				g *Inocybe*

Note: The last identifiable taxonomy levels were listed in the table.

### 3.6 Effects of temperature variables on phyllosphere fungal community assembly

Distance-based redundancy analysis (db-RDA) was performed to evaluate the effects of average temperature, accumulated temperature and host resistance phenotype on the assembly of the Norway spruce phyllosphere mycobiome. Average temperature and accumulated temperature explained a greater proportion of variation in fungal community composition than host resistance phenotype (partially resistant vs. susceptible) (Fig 6a).

**Fig 6:**
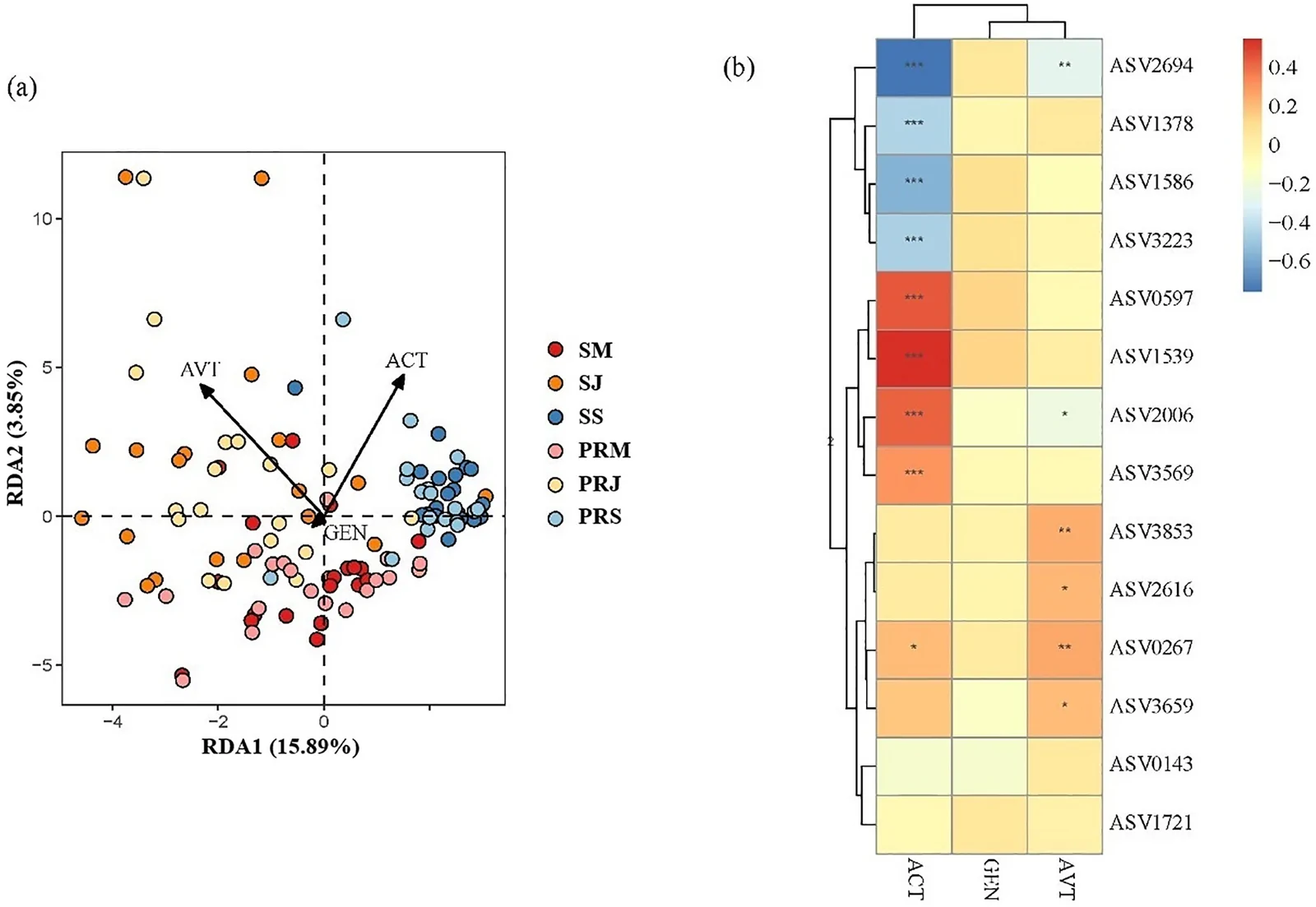
Driving factors for the Norway spruce phyllosphere mycobiome assemblage. (a) Redundancy analysis of Norway spruce phyllosphere microbiome and impact factors; (b) Spearman correlation of top 14 abundant fungal ASVs with impact factors. AVT: average temperature, ACT: accumulated temperature, GEN: genetic difference.

Spearman correlation analysis was subsequently performed to examine associations between these explanatory variables and the 14 most abundant fungal ASVs (Fig 6b). Nine ASVs were significantly correlated with accumulated temperature, whereas six ASVs were significantly correlated with average temperature. In contrast, none of the ASVs showed significant correlations with host resistance phenotype.

## 4. Discussion

### 4.1 Norway spruce phyllosphere mycobiome assemblage

Previous studies have shown that phyllosphere mycobiome assembly is governed by interacting ecological processes, including propagule dispersal, environmental filtering, host-mediated selection and stochastic ecological drift [45,46]. Consistent with this conceptual framework, our SourceTracker analysis indicated that more than 94% of the Norway spruce phyllosphere fungal community originated from non-seed sources, whereas only less than 1% could be attributed to seed-associated fungi. Therefore, this finding supports previous observations that phyllosphere fungal communities are assembled predominantly from environmental inoculum, including airborne spores, precipitation, and other external sources [45].

The limited overlap between seed and phyllosphere fungal communities suggests that most seed-associated fungi were unable to establish and persist in the phyllosphere after seedling emergence. This pattern likely reflects the distinct ecological conditions of the phyllosphere, where environmental filtering and host-mediated selection favour fungi adapted to the needle environment over those associated with seeds [47,48].

Although only a small proportion of phyllosphere taxa overlapped with the seed-associated fungal community, these early colonizers may nonetheless influence subsequent community assembly through priority effects and ecological succession [49,50]. However, this hypothesis was not directly evaluated in the present study. Interestingly, six fungal taxa were detected in both seed and phyllosphere samples of partially resistant genotypes. Among these, *Dissoconium nelumbonis* has been reported as an epiphyte on lotus leaves [51], *Dissoconium proteae* as an epiphyte on grape berries [52] and *Cystobasidium pinicola,* formerly known as *Rhodotonela pinicola,* as an endophyte of *Fraxinus excelsior* [53,54]. Whether these taxa contribute to phyllosphere establishment, microbial interactions, or host health in Norway spruce remains unknown and merits further investigation.

### 4.2 Genetic impact on the phyllosphere microbiome composition

Although host genetic effects on phyllosphere microbiomes have been widely investigated [8,9,31,32], how specific resistance traits influence fungal community assembly, and whether these effects vary seasonally, remain poorly understood. Our results showed that the phyllosphere mycobiome differed in richness, diversity and evenness between *Heterobasidion*-susceptible and partially resistant Norway spruce seedlings. However, these differences were almost entirely driven by samples collected in September, whereas no significant differences were detected in May or July. This indicates that the influence of host resistance phenotype on phyllosphere fungal communities is season dependent rather than constant throughout the growing season.

The season-dependent host effect observed in this study aligns with previous research showing strong temporal succession in phyllosphere microbial communities across the growing season [50,55]. This seasonal structuring of phyllosphere microbiomes has also been broadly synthesized across diverse plant systems, highlighting consistent patterns of temporal dynamics in both woody and herbaceous hosts [17]. Seasonal changes in host physiology, including growth cessation, cold acclimation, defence responses, and metabolite production, may strengthen host-mediated selection later in the growing season [56–58]. Likewise, genome-wide association studies in *Arabidopsis thaliana* have demonstrated that host defence-related loci contribute to phyllosphere microbiome assembly [59]. Together, these observations suggest that studies investigating host genetic effects on the phyllosphere microbiome should account for seasonal variation, as sampling at a single time point may underestimate or overlook genotype-dependent differences.

Host resistance phenotypes also influenced potential microbial interactions. Seasonal changes in co-occurrence network topology differed between susceptible and partially resistant seedlings, with the susceptible network becoming progressively more connected from May to September, whereas the partially resistant network remained comparatively stable. These differences coincided with shifts in fungal functional composition. In September, susceptible seedlings harboured a higher relative abundance of mutualistic symbionts and a lower abundance of saprotrophs than partially resistant seedlings, suggesting that host resistance phenotype may influence not only community composition but also potential ecological interactions among phyllosphere fungi. An experimental study of the phyllosphere microbiota has shown that microbial interactions can contribute to community structure and that changes in community composition can arise from direct interactions among microbial taxa [60]. However, co-occurrence patterns alone cannot establish direct biological interactions. The mechanisms underlying these seasonal differences observed here remain unresolved and therefore warrant further investigation.

At the taxonomic level, susceptible seedlings contained higher relative abundances of *Filobasidium* in May, and *Amphinema* in September than partially resistant seedlings. Species of *Filobasidium* possess diverse carbohydrate-active enzymes and have been associated with degradation of plant cell wall components and terpenoids [61–63], whereas *Amphinema* species are ectomycorrhizal fungi known to enhance nutrient acquisition and improve tolerance to environmental and biotic stresses [64–67]. Because needles were not surface sterilized prior to DNA extraction, the detection and increased relative abundance of *Amphinema* in September should be interpreted cautiously. Its occurrence in needle samples may reflect fungal propagules or DNA deposited on the needle surface rather than active epiphytic or endophytic colonization. Thus, the observed increase in *Amphinema* cannot be interpreted as evidence of phyllosphere establishment or activity. Previous studies have shown that host-related factors can shape needle-associated fungal communities in Norway spruce, with differences associated with host genotype [31] and current growth performance [32]. However, the ecological significance of these host-associated differences remains unclear. Future studies integrating surface-sterilized and non-sterilized samples with microscopy and culture-based approaches could help distinguish actively associated phyllosphere fungi from externally deposited fungal material. Such approaches would also help clarify whether these fungal communities influence host growth, performance, or susceptibility to disease.

### 4.3 Seasonal variation and temperature-associated changes in the phyllosphere mycobiome

Host genotype, temperature, and accumulated temperature associated with seasonal progression significantly influenced the assembly of the Norway spruce phyllosphere fungal microbiome. Both resistance phenotype and temperature-related variables (mean temperature and accumulated temperature) were significantly associated with variation in community composition (Fig 6 and S3 Fig), indicating that phyllosphere fungal assemblages are jointly structured by host and environmental drivers. Notably, seasonal progression had a pronounced effect on microbiome development, with host genotype effects being temporally constrained and detectable only in September in the present study (S3 Fig), suggesting that host-mediated filtering of the phyllosphere community intensifies later in the growing season.

In parallel, phyllosphere microbial communities are known to be highly sensitive to temperature variation, with multiple taxa showing strong correlations with temperature gradients across environments [68,69]. In addition, increased microbial richness has been reported in warmer environments under comparable host and stress conditions [4], further supporting the role of temperature as a key ecological filter in phyllosphere assembly.

In the present study, accumulated temperature showed stronger associations with the most abundant amplicon sequence variants (ASVs; relative abundance >0.1%), suggesting that long-term thermal conditions may better explain community structure than short-term temperature fluctuations. This pattern indicates that phyllosphere microbiome assembly is shaped by cumulative environmental exposure and extended host–microbe–environment interactions rather than instantaneous environmental states. Although phyllosphere communities are highly dynamic, microbial colonization, establishment, and functional expression likely require temporal windows to stabilize under changing environmental conditions [70,71].

Overall, phyllosphere fungal community composition in Norway spruce varied across the growing season and was associated with host resistance phenotype and temperature-related variables. However, seasonal and accumulated temperatures explained a greater proportion of variation in community structure than host genotype, highlighting the important drivers of environmental filtering in shaping phyllosphere assembly. In addition, only a small proportion of phyllosphere taxa overlapped with seed-associated fungal communities, although this overlap should be interpreted cautiously because the composition of seed-associated fungal communities may depend on the origin of the seed lot [72]**.** The observed seasonal variation is consistent with previous evidence that Norway spruce needle-associated fungal communities are influenced by environmental conditions as well as host-related factors [31,73]. Future studies incorporating seed-lot origin, additional environmental variables, host physiological traits, and experimental approaches will be required to clarify the relative contributions of seed-associated and environmental sources to phyllosphere microbiome assembly in Norway spruce.

### 4.4 Study limitations and future perspectives

A methodological consideration of this study is the use of rarefaction to standardize sequencing depth across samples. Rarefaction provided a common sequencing depth for comparative analyses and was supported by the observed sequencing-depth profiles, which indicated adequate sampling coverage at the selected depth. However, rarefaction involves random subsampling and consequently discards sequencing information from samples with greater read depth. This may reduce statistical power and sensitivity, particularly for analyses of community composition and low-abundance taxa. Nevertheless, the standardized approach facilitated consistent comparisons of fungal communities across genotypes and sampling periods. Future studies could complement these analyses using non-rarefied count data with appropriate normalization or compositional methods, particularly for beta-diversity and differential-abundance analyses.

A further limitation concerns the statistical analysis of fungal relative-abundance data. Although normality and homogeneity of variance were assessed before applying parametric tests, conventional parametric tests do not explicitly account for the compositional nature of microbiome data, in which the relative abundance of one taxon is inherently dependent on the abundances of other taxa. Future studies should consider compositionally aware statistical methods for differential-abundance analyses to strengthen inference. Together, these approaches would enhance the statistical resolution and interpretation of phyllosphere fungal community patterns in Norway spruce.

## Supporting information

S1 Fig Experimental design for seedling planting. (PDF)

S2 Fig Temperature during the sampling periods. (PDF)

S3 Fig Alpha diversity indices comparison between susceptible and partially resistant groups in May, July, and September. (PDF)

S1 Table Differentially abundant fungal taxa between susceptible and partially resistant genotypes across sampling periods based on Student’s *t*-tests. (XLSX)

S2 Table Identified genera and ASVs counts from Symbiotroph. (PDF)

S3 Table Student’s *t*-test comparison of the top 10 mycobiome genera between susceptible (SD) and partially resistant (PRD) genotypes. (PDF)

S4 Table Student’s *t*-test comparison of identified functions between susceptible and partially resistant seeds. (PDF)

Supplementary File R R markdown for analyses performed in R (RMD)
