# Supplementary material for: Host genotype, resistance status and seasonal temperature shape the Norway spruce phyllosphere mycobiome
Source: PLoS One. 2026 Oct 23;21(10):e0360930. doi: 10.1371/journal.pone.0360930 (PMC13693881; doi:10.1371/journal.pone.0360930)
Supplement: Supplementary file 1 [file pone.0360930.s001.pdf]

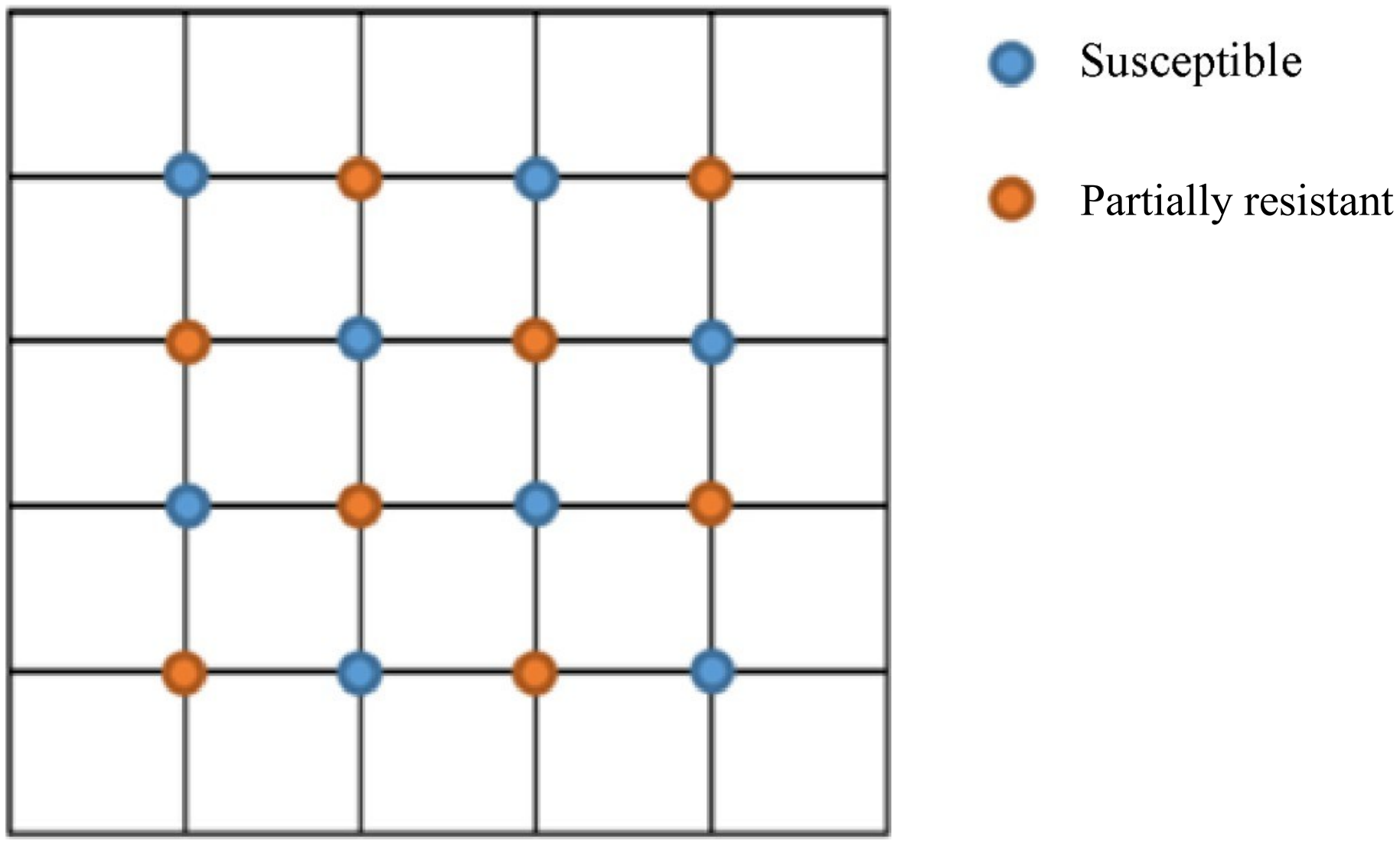
Susceptible
Partially resistant
