# Supplementary material for: Host genotype, resistance status and seasonal temperature shape the Norway spruce phyllosphere mycobiome
Source: PLoS One. 2026 Oct 23;21(10):e0360930. doi: 10.1371/journal.pone.0360930 (PMC13693881; doi:10.1371/journal.pone.0360930)
Supplement: Supplementary file 2 [file pone.0360930.s002.pdf]

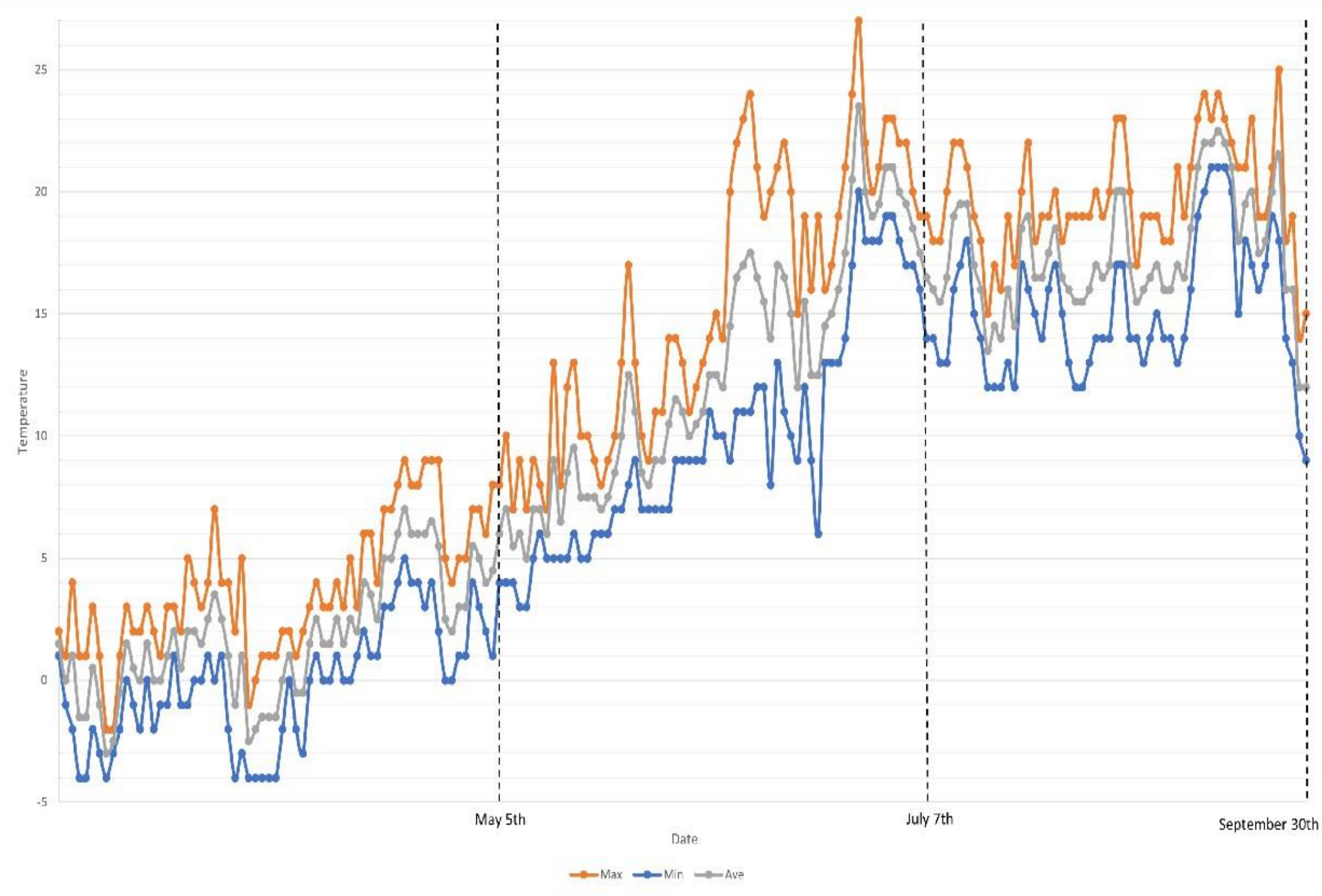

Temperature
25
20
15
10
5
0
-5
May 5th
July 7th
September 30th
Date
Max
Min
Ave
