# Supplementary material for: Host genotype, resistance status and seasonal temperature shape the Norway spruce phyllosphere mycobiome
Source: PLoS One. 2026 Oct 23;21(10):e0360930. doi: 10.1371/journal.pone.0360930 (PMC13693881; doi:10.1371/journal.pone.0360930)
Supplement: Supplementary file 3 [file pone.0360930.s003.pdf]

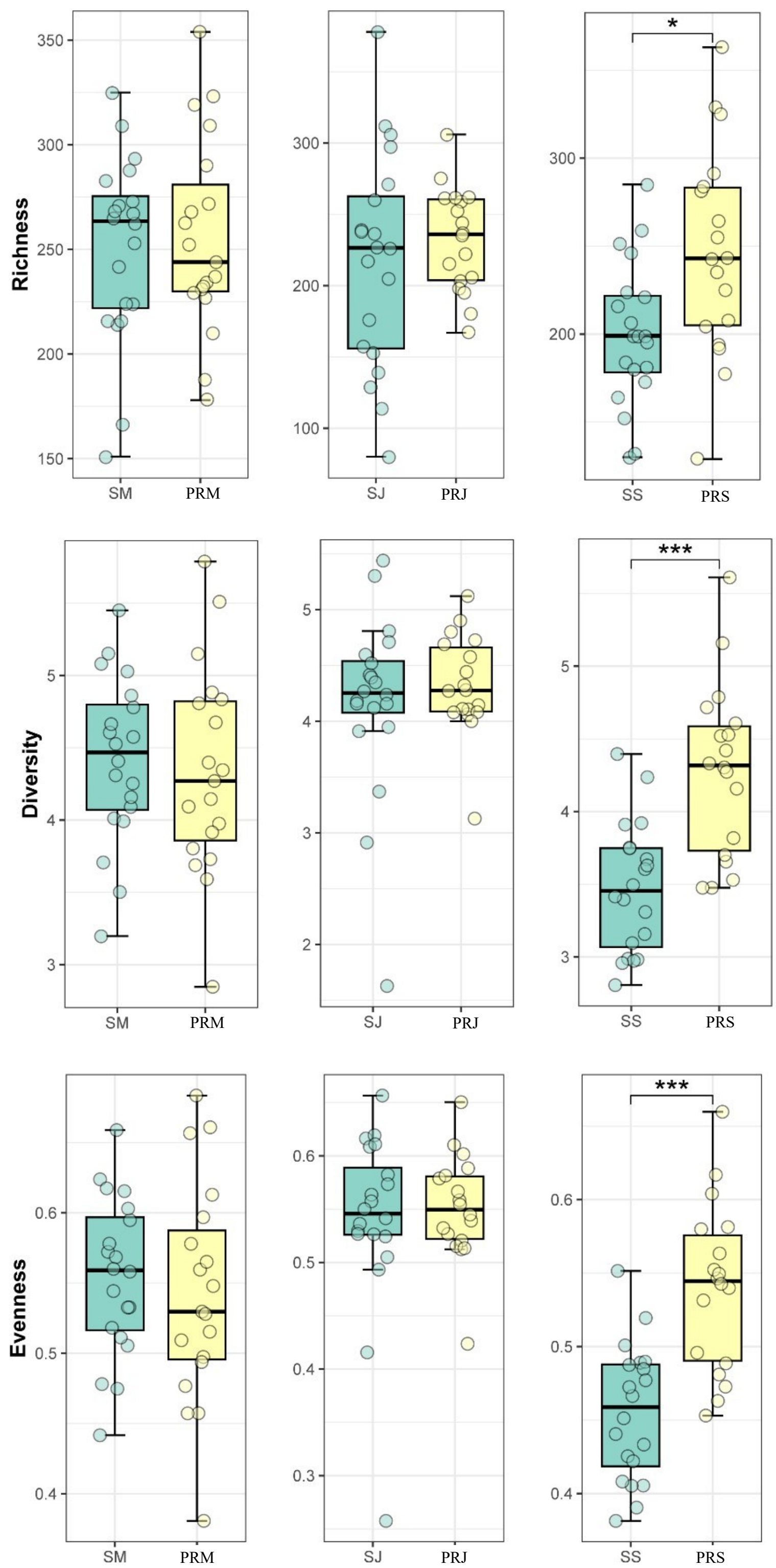

SM: Susceptible in May
SJ: Susceptible in July
SS: Susceptible in September

PRM: Partially resistant in May
PRJ: Partially resistant in July
PRS: Partially resistant in September
