# Supplementary material for: Host genotype, resistance status and seasonal temperature shape the Norway spruce phyllosphere mycobiome
Source: PLoS One. 2026 Oct 23;21(10):e0360930. doi: 10.1371/journal.pone.0360930 (PMC13693881; doi:10.1371/journal.pone.0360930)
Supplement: Supplementary file 4 [file pone.0360930.s005.pdf]

Trophic Mode	Genus	ASVs Sum
Symbiotroph	Amphinema	93
	Cadophora	20
	Inocybe	19
	Phialocephala	17
	Physcia	10
	Lecanora	7
	Rinodina	6
	Scoliciosporum	6
	Ballistosporomyces	3
	Cetraria	3
	Hypogymnia	3
	Melanohalea	3
	Stictidaceae_gen_Incertae_sedis	3
	Tylopilus	3
	Alatosessilispora	2
	Astraeus	2
	Lecythophora	2
	Phylliscum	2
	Polycauliona	2
	Teloschistaceae_gen_Incertae_sedis	2
	Trichomerium	2
	Xanthoria	2
	Acephala	1
	Athallia	1
	Camptophora	1
	Candelariella	1
	Coniozyma	1
	Fuscidea	1
	Geopora	1
	Melanomma	1
	Parmelia	1
	Pectenia	1
	Placynthiella	1
	Pseudevernia	1
	Rhizoplaca	1
	Tuckermanopsis	1
	Xylopsora	1
