# Supplementary material for: Host genotype, resistance status and seasonal temperature shape the Norway spruce phyllosphere mycobiome
Source: PLoS One. 2026 Oct 23;21(10):e0360930. doi: 10.1371/journal.pone.0360930 (PMC13693881; doi:10.1371/journal.pone.0360930)
Supplement: Supplementary file 5 [file pone.0360930.s006.pdf]

tax	group1	group2	p	p.signif
Hormonema	SD	PRD	0.472995	ns
Aureobasidium	SD	PRD	0.5236	ns
Sirococcus	SD	PRD	0.54067	ns
Inocybe	SD	PRD	0.386701	ns
Amphinema	SD	PRD	0.630801	ns
Perusta	SD	PRD	0.329753	ns
Vishniacozyma	SD	PRD	0.486616	ns
Kendrickiella	SD	PRD	0.479309	ns
Alternaria	SD	PRD	0.490761	ns
Sporobolomyces	SD	PRD	0.357024	ns
