# Supplementary material for: Host genotype, resistance status and seasonal temperature shape the Norway spruce phyllosphere mycobiome
Source: PLoS One. 2026 Oct 23;21(10):e0360930. doi: 10.1371/journal.pone.0360930 (PMC13693881; doi:10.1371/journal.pone.0360930)
Supplement: Supplementary file 6 [file pone.0360930.s007.pdf]

| tax | group 1 | group2 | p | p.signif |
|---|---|---|---|---|
| Algal Parasite | SD | PRD | 0.373901 | ns |
| Animal Endosymbiont | SD | PRD | 0.876543 | ns |
| Animal Pathogen | SD | PRD | 0.991505 | ns |
| Bryophyte Parasite | SD | PRD | 0.446655 | ns |
| Endosymbiont | SD | PRD | 0.373901 | ns |
| Epiphyte | SD | PRD | 0.530924 | ns |
| Ericoid Mycorrhizal | SD | PRD | 0.373901 | ns |
| Fungal Parasite | SD | PRD | 0.576247 | ns |
| Insect Parasite | SD | PRD | 0.373901 | ns |
| Leaf Saprotroph | SD | PRD | 0.768419 | ns |
| Lichen Parasite | SD | PRD | 0.410947 | ns |
| Lichenized | SD | PRD | 0.178779 | ns |
| Litter Saprotroph | SD | PRD | 0.365124 | ns |
| Plant Parasite | SD | PRD | 0.373901 | ns |
| Plant Pathogen | SD | PRD | 0.506754 | ns |
| Plant Saprotroph | SD | PRD | 0.481588 | ns |
| Soil Saprotroph | SD | PRD | 0.442826 | ns |
| Undefined Saprotroph | SD | PRD | 0.388758 | ns |
| Wood Saprotroph | SD | PRD | 0.797465 | ns |
| unassigned | SD | PRD | 0.923915 | ns |
| Endophyte | SD | PRD | 0.923149 | ns |
| Dung Saprotroph | SD | PRD | 0.24895 | ns |
| Ectomycorrhizal | SD | PRD | 0.306656 | ns |
